# Effects of corticotropin releasing factor (CRF) on sleep and temperature following predictable controllable and uncontrollable stress in mice

**DOI:** 10.3389/fnins.2015.00258

**Published:** 2015-07-30

**Authors:** Laurie L. Wellman, Linghui Yang, Larry D. Sanford

**Affiliations:** ^1^Sleep Research Laboratory, Department of Pathology and Anatomy, Eastern Virginia Medical SchoolNorfolk, VA, USA; ^2^West China Hospital of Sichuan UniversitySichuan, China

**Keywords:** corticotropin releasing factor, stress, predictability, controllability, escape learning, stress-induced hyperthermia

## Abstract

Corticotropin releasing factor (CRF) is a major mediator of central nervous system responses to stressors, including alterations in wakefulness and sleep. However, its role in mediating stress-induced alterations in sleep has not been fully delineated. In this study, we assessed the role of CRF and the non-specific CRF antagonist, astressin (AST), in regulating changes in sleep produced by signaled, escapable shock (SES) and signaled inescapable shock (SIS), two stressors that can increase or decrease sleep, respectively. Male BALB/cJ mice were surgically implanted with transmitters (DataSciences ETA10-F20) for recording EEG, activity and core body temperature by telemetry and a cannula for intracerebroventricular (ICV) microinjections. After baseline (Base) sleep recording, mice were presented tones (90 dB, 2 kHz) that started 5.0 s prior to and co-terminated with footshock (0.5 mA; 5.0 s maximum duration). SES mice (*n* = 9) always received shock but could terminate it by moving to the non-occupied chamber in a shuttlebox. Yoked SIS mice (*n* = 9) were treated identically, but could not alter shock duration. Training with SES or SIS was conducted over 2 days to stabilize responses. Afterwards, the mice received saline, CRF [0.4 μg (0.42 mM) or AST (1.0 μg (1.4 mM)] prior to SES or SIS. Sleep was analyzed over 20 h post-stress recordings. After administration of saline, REM was significantly greater in SES mice than in SIS mice whereas after CRF or AST, REM was similar in both groups. Total 20 h NREM did not vary across condition or group. However, after administration of saline and CRF, NREM episode duration was significantly decreased, and NREM episode number significantly increased, in SIS mice compared to SES animals. SES and SIS mice showed similar stress induced hyperthermia (SIH) across all conditions. These data demonstrate that CRF can mediate stress-induced changes in sleep independently of SIH, an index of hypothalamic-pituitary-adrenal axis activation.

## Introduction

Corticotropin releasing factor (CRF) is a major mediator of central nervous system responses to stressors (Koob and Bloom, 1985; Heinrichs et al., 1995; Koob, 1999; Koob and Heinrichs, 1999; Bakshi and Kalin, 2000; Deussing and Wurst, 2005) including alterations in wakefulness and sleep (González and Valatx, 1998; Chang and Opp, 2002). CRF antagonists attenuate behavioral responses to stress (e.g., Aloisi et al., 1999; Basso et al., 1999; Deak et al., 1999) whereas intracerebroventricular (ICV) administration of CRF can produce many of the signs associated with anxiety in humans, including increased wakefulness (Ehlers et al., 1986; Marrosu et al., 1990; Chang and Opp, 1998), increased activity, and an exaggerated startle response (Swerdlow et al., 1986; Heilig et al., 1994). In the absence of stressors, CRF contributes to the regulation of spontaneous waking (Opp, 1995, 1997; Chang and Opp, 1998, 2001).

CRF has been implicated in the control of rapid eye movement sleep (REM) (González and Valatx, 1997) and some authors have argued that CRF promotes REM. However, there actually have been few studies examining the role of CRF in regulating stress-induced alterations in sleep, and these have yielded conflicting data. This is exemplified with the work on restraint stress and sleep. For example, González and Valatx (1997) reported that the ICV administration of the broad CRF antagonist, αHelCRF (α-helical CRF9-41), prior to restraint stress at the beginning of the dark period prevented the subsequent increase in REM that can occur after restraint (e.g., Rampin et al., 1991; Bonnet et al., 1997; Meerlo et al., 2001), but did not alter spontaneous REM, NREM, or wakefulness in non-stressed animals. In contrast, Chang and Opp found no effect of restraint stress applied at the beginning of the dark period on subsequent sleep, and also found no effect of the non-specific CRF antagonist, astressin (AST), on sleep after restraint (Chang and Opp, 2002). There also are conflicting data on the effects of CRF on REM recovery after sleep deprivation. CRF antagonists administered during sleep deprivation reduce recovery REM in rats (González and Valatx, 1998) and mice (Kimura et al., 2010) whereas the repeated administration of CRF during the actual recovery period blocked the increase in REM in sleep-deprived humans (Schüssler et al., 2006).

Fear conditioning with inescapable shock (IS), an uncontrollable stressor, and the presentation of fearful contexts and cues associated with IS are followed by significant reductions in REM that occur in the first few hours after exposure (Sanford et al., 2003a,b,c; Pawlyk et al., 2005). In mice trained with IS, ICV administration of CRF enhances the reduction in REM following fearful contexts whereas ICV administration of AST attenuates fear-induced reductions in REM (Yang et al., 2009). By comparison, training with escapable shock (ES), and reminders of ES, can produce significant enhancements in post-stress REM (Sanford et al., 2010). Microinjections of either saline or AST prior to ES training produce similar, significant enhancements in post-stress REM relative to a non-shocked handling control condition whereas the increases in REM are blocked by pre-training treatment with CRF (Yang et al., 2011b). Direct comparisons of the effects of manipulating CRF on alterations in sleep associated with controllable and uncontrollable stress have not been made; however, the results to date are consistent with a suppressing effect of CRF on REM after exposure to stress.

In summary, studies administering antagonists prior to or during the presentation of stressors or sleep deprivation have led to conclusions that CRF can promote REM whereas studies administering CRF in stress paradigms suggest that that it can suppress REM. In the current study, we compared the effects of CRF and AST on stress-induced alterations in sleep in mice trained with auditory-signaled variants of ES and IS (SES and SIS, respectively), which also produce equivalent physical stress but directionally different alterations in REM (Yang et al., 2011a). We concurrently examined stress-induced hyperthermia (SIH), an increase in core body temperature induced by both physiological and psychological stress (Vinckers et al., 2009), as a measure of the acute stress response. This study design enabled us to determine the effects of CRF and AST on stressors that produce predictable increases and decreases in REM.

## Methods

### Subjects

Male BALB/cJ mice (*n* = 18) were obtained from Jackson Laboratory, Bar Harbor, Maine. The mice weighed 20–25 g at arrival. Animals were individually housed with food and water available *ad libitum*. The mouse colony room was kept on a 12:12 light-dark cycle and ambient temperature was maintained at 24 ± 1.5°C. Throughout the experimental procedures, measures were taken to minimize unnecessary pain and discomfort of the animals.

### Surgery

All mice were implanted intraperitoneally with telemetry transmitters (DataSciences ETA 10-F20) for recording EEG, body temperature and activity as previously described (Tang and Sanford, 2002). EEG leads from the transmitter body were led subcutaneously to the head, and the free ends were placed into holes drilled in the dorsal skull to allow recording cortical EEG. In the same surgery, the mice were stereotaxically implanted with a cannula to allow ICV microinjections. A hole was drilled in the skull 1.00 mm lateral and 0.5 mm posterior to Bregma and the tip of a 26-gauge stainless steel infusion cannula was placed 2.00 mm below the skull surface into the right ventricle. The cannula was secured to the skull with dental cement and a stylus was inserted to maintain patency. All surgeries were performed under aseptic conditions and with the mice under isoflurane (as inhalant: 5% induction; 1–2% maintenance) anesthesia. Ibuprofen (30 mg/kg, orally) was continuously available in each animal's drinking water for 24–48 h preoperatively and for a minimum of 72 h postoperatively to alleviate potential postoperative pain. Antibiotics (gentamicin 5–8 mg/kg and Procaine penicillin 100,000 IU/kg) were given subcutaneously preoperatively to prevent infection. Dexamethazone [0.4 mg (0.2 ml total dosage)] was administered subcutaneously preoperatively to reduce brain swelling. All procedures were conducted in accordance with the National Institutes of Health Guide for the Care and Use of Experimental Animals and were approved by Eastern Virginia Medical School's Animal Care and Use Committee.

### Baseline and control recordings

The mice were housed and studied in the same room. Cages and bedding were changed 2 days prior to recording onset for each phase of the experiment and then not disturbed until that phase was complete.

The mice were allowed a post-surgery recovery period of 19–20 days prior to beginning the experiment. Undisturbed baseline (Base) recordings were then obtained for 2 days. Afterwards, the mice were habituated to two daily sessions of the handling procedures needed for administering microinjections. Sleep also was recorded following a 30 min exposure to a novel chamber [an enclosure of approximately the same dimensions as the shock chambers (20 × 40 × 30 cm)] with an open top and walls and floor constructed of clear Plexiglas™. This recording session controlled for handling and exposure to a non-cage environment and was used as a handling control (HC) for comparisons across treatment conditions.

### Signaled escapable and inescapable shock (SES/SIS) training procedures

The mice were randomly assigned to either SES (*n* = 9) or SIS (*n* = 9) conditions. Training was conducted in a shuttlebox (Model E10-15SC, Coulbourn Instruments, Whitehall, PA) consisting of two chambers divided by a guillotine door. Opening and closing of the guillotine door, as well as the tone (90 dB, 2 kHz, 10 s maximum) and footshock (0.5 mA; 5.0 s maximum) administration were controlled by Coulbourn Graphic State (GS) software (version 2.1). Electric footshock was produced via Coulbourn Precision Regulated Animal Shockers and administered via grid floors of a shuttlebox. Training started between the third and fourth hour after lights on. The entire training procedure was of approximately 30 min duration. For the first 5 min (pre-shock period), the mice were allowed to freely explore the shuttlebox followed by presentation of 20 shocks. The tone was presented 5 s prior to the footshock to signal its onset. When footshock was administered, the SES mice were then able to move through the door to the unoccupied chamber. This movement caused interruption of photo-beam sensors which was detected by GS software and terminated shock presentation and tone. If the animal did not move to the unoccupied chamber, the shock and tone co-terminated after 5 s. Footshock for SES mice was *escapable*, but not avoidable, for movement prior to footshock onset did not prevent shock presentation. SES and SIS mice were simultaneously trained as yoked pairs such that SIS mice received identical amounts of shock as a mouse in the SES group, but could not alter shock duration by their actions. After the final shock, the animals remained undisturbed in the shuttlebox for 5 additional min (post-shock period) and then were returned to their home cages.

The mice received 2 days of 20 (1.0 min intervals) tone-shock pairings without drug administration to stabilize responses. On four subsequent days, the mice were administered saline (SAL), CRF or AST prior to SES or SIS. The experiment was pseudo-counterbalanced in that the series of CRF microinjections were completed before starting the series of AST microinjections. SAL was administered such that approximately half the mice received SAL on each of the four training days. Each shock presentation day with injection was separated by 1 week.

### Drugs and microinjections

After recovery from surgery, ICV location of the cannula was verified with administration of angiotensin (200 ng in 0.2 μl ICV) and observation for drinking. A positive angiotensin-induced drinking response was shown by all mice included in the study.

CRF and AST [cyclo(33)[D-Phe12,N1e21,38,Glu30,Lys33] h/rCRF(12-41)] were obtained in powder form from Sigma-Aldrich (St. Louis, MO) and were diluted to the desired concentrations in pyrogen-free SAL. Concentrations [CRF 0.4 μg (0.42 mM); AST: 1.0 μg (1.4 mM)] used in this experiment were based on dosages used in previous studies (Sanford et al., 2008; Yang et al., 2009).

For microinjections, an injection cannula (33 ga.), which projected 1.0 mm beyond the tip of the implanted guide cannula, was inserted and secured in place. The injection cannula was connected to lengths of polyethylene tubing that in turn were connected to a 5.0 μl Hamilton syringe. The injection cannula and tubing had been pre-filled with the solution to be injected. The solutions in a volume of 0.2 μl were slowly infused over 1 min and administered 15 min prior to beginning shock training.

### Sleep recording and determination of sleep state

Sleep and wakefulness of the animals were monitored in the colony room. Recording started at the fifth hour after lights on. The transmitters for the mice were activated with a magnetic switch, and their home cages were placed on a telemetry receiver (Model RPC-1, Data Sciences International). When the animals were not on study, the transmitters were inactivated. Signals from the transmitter were detected by the receiver, and were processed and saved by Data Sciences International software for subsequent visual scoring. Sleep and wakefulness were determined by a trained observer in 10-s epochs using SleepWave software (Biosoft Studio). Each epoch was scored either as NREM, REM, active wakefulness (AW), or quiet wakefulness (QW), based on EEG and gross whole body activity as previously described (Tang and Sanford, 2002). Twenty hours uninterrupted sleep recordings were collected for Base and after each experimental session.

### Data analyses

Data were analyzed using Sigmaplot 12 software (Systat Software, Inc. San Jose, CA). The data were primarily analyzed with two-way mixed factorial ANOVA procedures (Group X Treatment Day with repeated measures on Treatment Day. When appropriate, *post-hoc* comparisons among means were conducted using Holm-Sidak tests. Differences were considered significant at *p* < 0.05.

## Results

Amounts of REM, NREM and total sleep did not differ across the two SAL recording days. We therefore collapsed data for these 2 days and made comparisons across Base, HC, SAL, CRF, or AST prior to shock training. Analyses were conducted on the total 20 h recordings and on the 8 h light and 12 h dark periods.

### Effects of CRF and AST on stress-induced alterations in REM

#### Total REM and REM percentage

**Figure 1:**
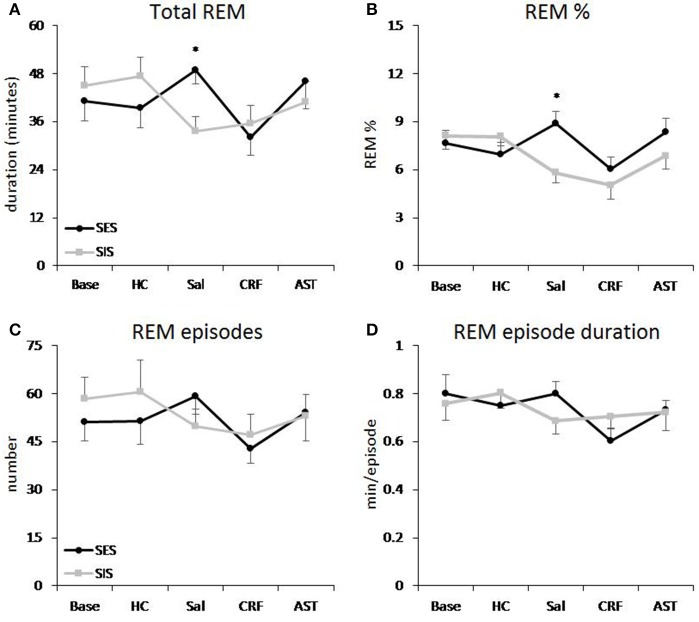
**REM sleep plotted as 20 h totals for baseline (Base), handling control (HC) and for days on which the mice received ICV injections of saline (SAL), corticotropin releasing factor (CRF) or astressin (AST) prior to signaled, escapable shock (SES) or to signaled, inescapable shock (SIS) training**. **(A)** Total REM; **(B)** REM % (total REM/total sleep time × 100%); **(C)** REM episodes; **(D)** average REM episode duration. Comparisons of SES (*n* = 9) and SIS (*n* = 9): ^*^p < 0.05. Values are means ± SEM.

The ANOVA for total 20 h REM amounts revealed a significant Treatment Day effect [*F*_(4, 64)_ = 2.85, *p* < 0.03] and a Group X Treatment Day interaction [*F*_(4, 64)_ = 3.55, *p* < 0.02]. *Post-hoc* comparisons across Treatment Days revealed a significant difference in REM between the SES and SIS groups only on the SAL day (Figure 1A). Amounts of REM during Base, and on the HC, CRF and AST recording days did not significantly differ between groups. Thus, administration of either CRF or AST reduced or eliminated the differences normally seen between mice trained with SES and SIS, i.e., CRF reduced REM in the SES mice to levels seen in the SIS mice and AST attenuated the reduction in REM in the SIS mice to levels that were not significantly different from those in the SES mice. The difference between groups on the SAL day was found primarily during the dark period which was also characterized by a significant Group X Treatment Day interaction [*F*_(4, 64)_ = 2.98, *p* < 0.03] and similar findings across Treatment Days (Table 1). The analysis for light period total REM found a significant Treatment Day effect [*F*_(4, 64)_ = 5.67, *p* < 0.01]. REM was reduced on the CRF Treatment Day compared to Base, HC, and AST (Table 1), but there were no significant differences between groups.

**Table 1:** **Selected REM sleep parameters for the 8 h light period and 12 h dark period during baseline (Base), handling control (HC), and for days on which the mice received ICV injections of saline (Sal), corticotropin releasing factor (CRF) or astressin (AST) prior to training with signaled, escapable shock (SES) or signaled, inescapable shock (SIS)**.

		**Base**	**HC**	**Sal**	**CRF**	**AST**
**LIGHT PERIOD**						
REM total	SES	13.4±2.4	15.5±2.5	12.6±2.0	5.7±1.6^+^^#^^∧^	15.2±4.9
	SIS	16.4±2.6	16.2±2.1	9.1±1.7	9.0±2.6^+^^#^^∧^	11.7±2.4
REM %	SES	6.1±0.8	5.7±0.7	5.6±0.8	2.9±0.7^+^^#^^¤^^∧^	5.7±1.4
	SIS	6.6±0.9	6.11±0.6	3.5±0.6	2.6±0.5^+^^#^^¤^^∧^	4.3±0.8
REM episodes	SES	11.5±2.9	10.5±1.4	9.8±1.5	4.9±1.1^#^	10.6±2.8
	SIS	12.5±2.1	11.9±2.1	7.8±1.3	6.8±1.5^#^	9.8±2.2
REM duration	SES	0.7±0.07	0.72±0.07	0.65±0.07	0.46±0.10^+^^#^	0.59±0.06
	SIS	0.67±0.05	0.77±0.07	0.58±0.05	0.58±0.06^+^^#^	0.64±0.13
**DARK PERIOD**						
REM total	SES	27.8±4.8	24.0±3.6	36.4±3.1^*^	26.4±4.0	30.9±3.9
	SIS	28.6±3.4	31.2±3.3	24.6±2.0	26.5±2.9	29.3±4.0
REM %	SES	8.3±1.1	8.0±1.0	11.1±0.9^*^	7.6±0.9	10.1±0.9
	SIS	9.5±0.9	9.6±0.8	7.7±0.8	6.6±1.4	8.9±1.2
REM episodes	SES	15.3±3.2	10.1±1.8	13.2±1.2	11.0±1.6	11.0±1.6
	SIS	11.1±1.1	12.3±2.0	11.4±1.2	11.1±1.5	11.2±1.2
REM duration	SES	0.87±0.1	0.78±0.06	0.90±0.06	0.70±0.04	0.83±0.06
	SIS	0.82±0.08	0.83±0.07	0.76±0.07	0.79±0.05	0.78±0.06

*Comparisons between SES (n = 9) and SIS (n = 9)*:

* *p < 0.05*;

*Comparisons to Base*:

+ *p < 0.05*;

*Comparisons to HC*:

# *p < 0.05*;

*Comparisons to Sal*:

¤ *p < 0.05*;

*Comparisons to AST*:

∧ *p < 0.05*.

*Values are means ± SEM. Total REM and REM Duration are reported in minutes; REM% was calculated as total REM/total sleep time × 100%, and REM Episodes are reported as number of episodes*.

A similar Main Effect for Treatment Day [*F*_(4, 64)_ = 4.92, *p* < 0.01] and a Group X Treatment Day interaction [*F*_(4, 64)_ = 3.92, *p* < 0.01] were found for the analyses of total 20 h REM percentage (of total sleep time) and a significant interaction for dark period REM percentage [*F*_(4, 64)_ = 3.45, *p* < 0.02]. Differences between SES and SIS mice also were found only on the SAL Treatment Days (Figure 1B, Table 1). As with total REM amounts, the analysis for the light period did reveal any Group differences, though there was a significant Treatment Day effect [*F*_(4, 64)_ = 10.22, *p* < 0.001]. REM percentage was reduced on the CRF Treatment Day compared to Base, HC, SAL, and AST (Table 1).

#### Number of REM episodes and REM episode duration

There were no significant differences in number of REM episodes (Figure 1C) or REM episode duration (Figure 1D) in the analyses of the 20 h recording period. However, there was a significant Treatment Day effect for number of REM episodes during the light period [*F*_(4, 64)_ = 4.41, *p* < 0.01] and the *post-hoc* analysis found significantly reduced REM episodes on the CRF day compared to the Base day (Table 1). There also was a significant Treatment Day effect for REM episode duration during the light period [*F*_(4, 64)_ = 3.71, *p* < 0.01] and the *post-hoc* analysis found significantly reduced REM episode duration on the CRF day compared to HC (Table 1). No other comparisons were significant.

### Effects of CRF and AST on stress-induced alterations in NREM, total sleep time, and wakefulness

#### NREM and total sleep time

**Figure 2:**
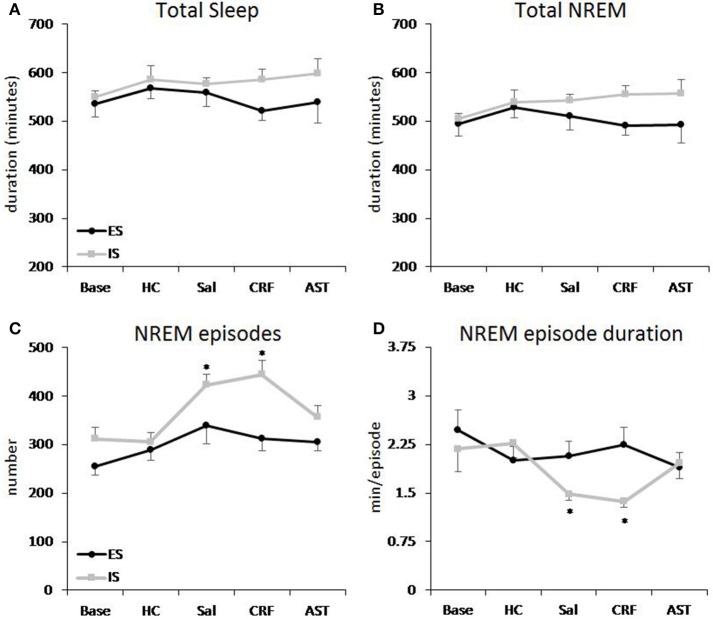
**Total sleep and amounts of NREM sleep plotted as 20 h totals for baseline (Base), handling control (HC) and for days on which the mice received ICV injections of saline (SAL), corticotropin releasing factor (CRF) or astressin (AST) prior to signaled, escapable shock (SES) or to signaled, inescapable shock (SIS) training**. **(A)** Total sleep; **(B)** Total NREM sleep; **(C)** NREM episodes; **(D)** average NREM episode duration. Comparisons of SES (*n* = 9) and SIS (*n* = 9): ^*^p < 0.05. Values are means ± SEM.

Total 20 h TST (Figure 2A) and NREM amounts (Figure 2B) did not vary across condition or group. However, there was a difference across Treatment Days for light period NREM [*F*_(4, 64)_ = 3.44, *p* < 0.02] and TST [*F*_(4, 64)_ = 4.15, *p* < 0.01]. Light period NREM was reduced on the CRF day compared to the HC day and light period TST was reduced on the CRF day compared to the HC and AST Treatment Days (Table 2). No other comparisons were significant.

**Table 2:** **Total sleep, NREM and wake parameters for the 8 h light period and 12 h dark period during baseline (Base), handling control (HC), and for days on which the mice received ICV injections of saline (Sal), corticotropin releasing factor (CRF) or astressin (AST) prior to training with signaled, escapable shock (SES) or signaled, inescapable shock (SIS)**.

		**Base**	**HC**	**Sal**	**CRF**	**AST**
**LIGHT PERIOD**						
Total sleep	SES	211.4±19.7	269.3±21.0	231.5±19.2	182.8±14.2^#^^∧^	239.7±24.1
	SIS	250.8±20.6	259.9±16.6	254.3±10.9	241.1±7.6^#^^∧^	270±16.9
NREM total	SES	198±18.0	253.8±19.7	218.2±18.7	177.1±13.1^#^	224.6±20.8
	SIS	234.4±19.3	243.7±15.0	245.2±10.0	234.9±7.5^#^	258.3±16.3
NREM episodes	SES	116.9±16.3	1.22±11.9	159.4±14.8^*^	133.6±18.4^*^	140.6±11.2
	SIS	150.9±13.8	143.6±10.8	192.9±13.4	198.7±13.0	168.7±12.8
NREM duration	SES	1.70±0.18	1.86±0.22	1.26±0.15^#^	1.44±0.24^#^	1.56±0.22
	SIS	1.49±0.19	1.53±0.16	1.10±0.06^#^	1.12±0.10^#^	1.36±0.12
Active wake	SES	132.8±20.0	96.3±17.0	111.6±18.9	134.7±21.2	130.5±22.0
	SIS	89.1±11.7	99.9±11.7	85.8±11.2	91.2±6.5	89.9±14.1
Quiet wake	SES	135.8±9.7^#^^∧^	114.5±9.5	136.9±12.8^#^^∧^	162.6±13.2^#^^∧^	109.7±7.1
	SIS	140.1±14.6^#^^∧^	120.3±8.3	139.9±4.7^#^^∧^	147.7±6.4^#^^∧^	120.1±7.9
**DARK PERIOD**						
Total sleep	SES	323.8±26.9	298.7±20.5	328.6±18.0	339.6±26.0	299.6±25.4
	SIS	299.8±21.2	326.2±15.9	323±9.5	344.9±21.2	328.3±16.6
NREM total	SES	296±23.7	274.7±18.7	292.2±17.0	313.2±23.8	268.7±22.4
	SIS	271.2±19.0	295±14.8	298.5±10.5	320.7±17.4	298.9±15.1
NREM episodes	SES	139.1±9.9	166.8±15.4	179.5±24.4^*^	179.3±12.8^*^	164.9±18.2
	SIS	161.3±15.4	162.3±17.3	230.3±12.2	245.8±19.4	188.3±15.0
NREM duration	SES	1.97±0.31	1.23±0.06	1.36±0.16	1.36±0.23	1.02±0.18
	SIS	1.75±0.22	1.33±0.11	1.12±0.13	1.23±0.12	1.14±0.22
Active wake	SES	197.8±13.1	209.1±25.9	203.2±28.1	168.8±20.1	233.2±32.7
	SIS	212.3±29.4	195.7±16.9	182.3±11.2	156.7±13.8	189.1±18.9
Quiet wake	SES	198.4±17.3	212.2±14.1	188.2±13.7	211.6±19.2	187.2±17.9
	SIS	207.9±16.7	198.1±11.0	214.6±7.8	218.4±13.1	202.7±11.5

*Comparisons between SES (n = 9) and SIS (n = 9)*:

* *p < 0.05*.

*Comparisons to Base: ^+^p < 0.05*.

*Comparisons to HC*:

# *p < 0.05*.

*Comparisons to AST*:

∧ *p < 0.01*.

*Values are means ± SEM. Total Sleep, NREM Total, NREM Duration, Active Wake, and Quiet Wake are reported in minutes. NREM Episodes are reported as number of episodes*.

The analysis of NREM episodes for the 20 h recording period (Figure 2C) revealed significant effects for Group [*F*_(1, 16)_ = 10.305, *p* < 0.01] and Treatment Day [*F*_(4, 64)_ = 8.483, *p* < 0.001]. This effect was primarily due to the increased number of NREM episodes in the SIS mice compared to the SES mice on the SAL and CRF treatment days compared to Base and HC. Virtually identical results were found for the analyses of the light [Group (*F*_(1, 16)_ = 7.949, *p* < 0.02) and Treatment Day [*F*_(4, 64)_ = 5.504, *p* < 0.001)] and dark [Group (*F*_(1, 16)_ = 4.973, *p* < 0.02] and Treatment Day [*F*_(4, 64)_ = 6.567, *p* < 0.001)] periods (Table 2).

There was a main effect for Treatment Day in the analysis of the NREM episode duration (Figure 2D) for the total 20 h recording period [*F*_(4, 64)_ = 7.669, *p* < 0.001). NREM episode duration was reduced on the SAL and CRF treatment days compared to Base and to the HC Treatment Day. There also was a significant Treatment Day effect during the light period [*F*_(4, 64)_ = 5.588, *p* < 0.001]; however, the reductions in NREM episode duration for the SAL and CRF treatment days were significantly reduced only compared to the HC treatment day (Table 2).

The analysis of NREM episode duration for the dark period revealed a significant effect for Treatment Day [*F*_(4, 64)_ = 6.610, *p* < 0.011] and a significant Group X Treatment Day [*F*_(4, 64)_ = 2.569, *p* < 0.05]. The duration of dark period NREM episodes were significantly reduced in the SIS mice than in the SES mice on the SAL and CRF treatment days (Table 2).

#### Active waking and quiet waking

**Figure 3:**
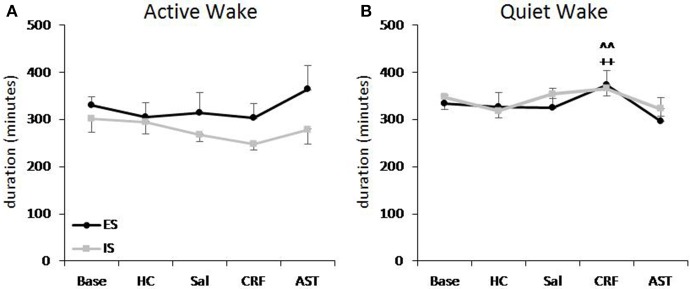
**Total amounts of active waking (A) and quiet waking (B) plotted as 20 h totals for baseline (Base), handling control (HC) and for days on which the mice received ICV injections of saline (SAL), corticotropin releasing factor (CRF), or astressin (AST) prior to signaled, escapable shock (SES, ***n*** = 9) or to signaled, inescapable shock (SIS, ***n*** = 9) training**. Comparisons of CRF to HC: ++, *p* < 0.01. Comparisons of CRF to AST: ^∧∧^*p* < 0.01. Values are means ± SEM.

Total 20 h amounts of active waking did not significantly differ across groups or conditions (Figure 3A). By comparison, the analysis of quiet waking revealed a significant Treatment Day effect [*F*_(4, 64)_ = 5.13, *p* < 0.01]. Quiet waking was greater on the CRF day than on the HC or AST treatment days (Figure 3B). There also was significant effect for Quiet waking in the light period [*F*_(4, 64)_ = 9.274, *p* < 0.001]. Light period quiet waking was significantly greater during Base and on the SAL and CRF treatment days than on the HC or AST treatment days (Table 2).

### Effects of CRF and AST on stress-induced alterations in core body temperature

**Figure 4:**
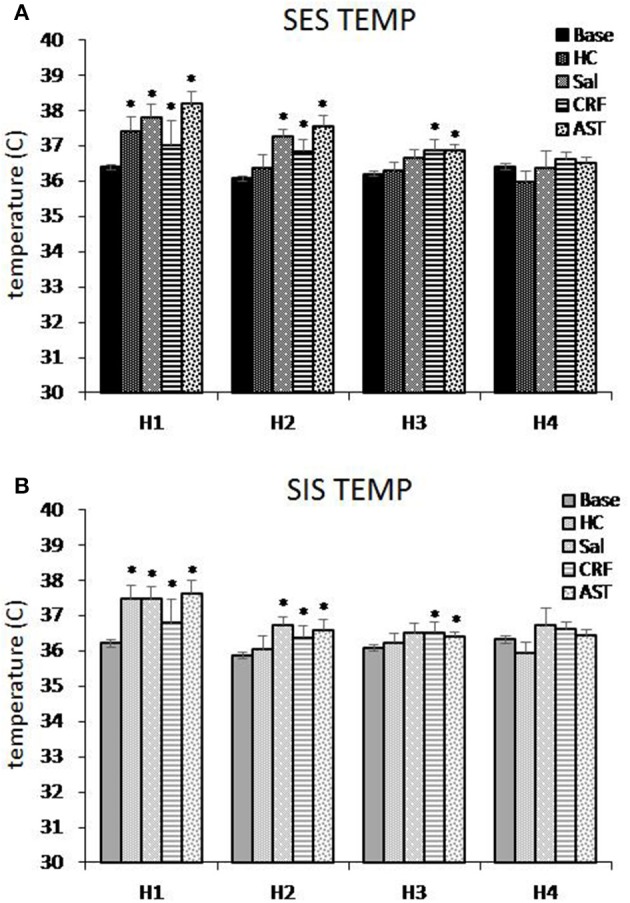
**Average core body temperature plotted hourly for the first 4 h of the 20 h recording period for mice trained with signaled, escapable shock (SES, A) or signaled, inescapable shock (SIS, B) training**. Average temperature is shown for baseline (Base), handling control (HC), and for ICV microinjections of saline (SAL), corticotropin releasing factor (CRF), and astressin (AST) prior to SES (*n* = 9) or SIS (*n* = 9). Comparisons to Base: ^*^p < 0.05. Values are means ± SEM.

We examined changes in core body temperature hourly across the first 4 h of recording to assess potential differences in SIH produced by SES (Figure 4A) and SIS (Figure 4B) and the effects of CRF and AST. The ANOVAs revealed a main effect for Treatment Day for h 1 [*F*_(4, 64)_ = 9.083, *p* < 0.001]; h 2 [*F*_(4, 64)_ = 11.721, *p* < 0.001] and h 3 [*F*_(4, 64)_ = 3.013, *p* < 0.03]. In h 1, compared to Base, core body temperature was significantly increased for HC, SAL, CRF, and AST. In h 2, temperature in the HC treatment did not differ significantly from that in Base, but remained elevated in SAL, CRF, and AST. There also was a Group effect in h 2 [*F*_(1, 16)_ = 4.505, *p* < 0.05] and temperature was greater in the SES mice (36.83°C) than in the SIS mice (36.33°C). In h 3, temperature remained significantly higher in the CRF and AST treatment condition compared to Base whereas the difference for SAL did not reach significance (*p* = 0.07). There were no significant differences between SES and SIS, and by h 4, temperature had returned to Base levels in all conditions.

## Discussion

The present results support previous studies (Sanford et al., 2010; Yang et al., 2011a,b) demonstrating that signaled and non-signaled controllable and uncontrollable stress are followed by directionally different alterations in REM in the post-stress period even though the acute stress response (as indicated by SIH) is similar. The results also indicate that centrally acting CRF is a significant factor in the reduction in REM that follows uncontrollable stress. This conclusion is supported by findings that differences in REM between SES and SIS trained mice are reduced by ICV administration of CRF and AST, with CRF resulting in reduced REM in both groups and with AST attenuating the reduction in REM in the SIS group. SIS, but not SES, also produced significant changes in NREM architecture, but not total amount, which were ameliorated by antagonizing CRF. The increases in the number of NREM episodes and decreases in NREM episode duration after SIS suggests more fragmented sleep even though the amount of sleep did not change. Thus, our results indicate a strong role for CRF in mediating stress-induced reductions in REM and a likely role for CRF in disrupting sleep continuity after certain types of stressors. Despite significant differences in REM, SIH was similar for SIS and SES treatments with and without co-administration of CRF or AST. These data suggest that central CRF may play a minimal role in modulating stress-induced alterations in temperature in this model or that sleep may be more sensitive to perturbations of the central CRF system than are peripheral indices of the stress response.

### Stressor controllability and predictability and sleep

Whether an animal perceives stressors as controllable and/or predictable appears to be an important factor in the effects of stress (Abbott et al., 1984; Adell et al., 1988). Both controllability and predictability can significantly influence post-stress sleep, and there appears to be the potential for interactions between the type of stress-related information available to the animal, its processing of that information, and the subsequent effects on sleep. In previous work, we demonstrated that auditory cues that predict shock can modify its effects on sleep and, contrary to contexts associated with non-signaled escapable footshock, do not produce conditioned increases in REM (Yang et al., 2011a). Learning the appropriate escape response (control) is also necessary for increases in REM (Machida et al., 2013).

The state of the animal at the time of stress also appears to be important. The brief manual restraint necessary for conducting microinjections is itself a stressor that can impact the animals and significantly alter subsequent sleep (Tang et al., 2007). It can also influence the effects of other stressors on sleep (Tang et al., 2007). For example, in rats, 5 min of manual restraint administered prior to 20 inescapable footshocks prevented the decrease in light period REM normally observed when inescapable footshock is presented without prior restraint. In a previous study reporting on the effects of SIS and SES without prior microinjections, we found an increase in NREM after shock training in SIS mice compared to SES mice (Yang et al., 2011a) that we did not see in the present study. This difference may have been due to the handling needed to complete the microinjections, as all other conditions were virtually the same across studies. Thus, our results should be interpreted in the context of the effects of CRF and AST on two interacting stressors, brief restraint and SES or SIS, as well as the complex emotions and memories and potential learning associated with the two conditions.

### CRF and the regulation of sleep and arousal

Several lines of existing data indicate that CRF is a significant regulator of stress-induced alterations in sleep and arousal; however, its actual role remains to be clarified. Studies administering antagonists prior to or during the presentation of stressors (González and Valatx, 1997) or sleep deprivation (González and Valatx, 1998; Kimura et al., 2010) have led to the conclusion that CRF can promote REM. Interestingly, there also is evidence that systemic administration of some CRF antagonists can inhibit spontaneous REM in adult (Ahnaou et al., 2012) and neonatal (Liu et al., 2010) rats, which can be considered supportive of this conclusion. However, studies that administered CRF prior to stress (Yang et al., 2009, 2011b) or during the recovery period after sleep deprivation (Schüssler et al., 2006) indicate that CRF can be inhibitory to REM. Under non-stress conditions, we also found that ICV administration of AST decreased wakefulness and increased REM in stress-responsive BALB/cJ mice, but not in less-responsive C57BL/6J mice, whereas CRF decreased REM in both strains (Sanford et al., 2008). Both of these studies can be considered consistent with the hypothesis that CRF has a suppressing effect on REM.

Resolving the discrepancies regarding the role of CRF in mediating the effects of stress on REM will likely require considering the relationship between the time course of the CRF stress response and that of stress-induced alterations in sleep and arousal. Many indices of stress including corticosterone (Veening et al., 2004; van Bogaert et al., 2006), SIH (Veening et al., 2004; van Bogaert et al., 2006), and activation of the hypothalamic paraventricular nucleus and other stress reactive regions in the brain (Grahn et al., 1999; Coco and Weiss, 2005; Liu et al., 2009) return to pre-stress levels relatively shortly after termination of an acute stressor. By comparison, alterations in sleep can occur several hours into the post-stress period. Studies administering an antagonist prior to presenting the stressor simply may have prevented or attenuated the initial CRF response which then altered the subsequent post-stress increase in REM that is seen with many stressors. Whether CRF is acting centrally or peripherally may also have consequences for its potential effects on REM in stress or non-stress conditions. This is suggested by findings that elevations of corticosterone after CRF is given ICV appear to be able to reduce REM even in CRF receptor 1 knockout mice (Romanowski et al., 2010).

The complexity of trying to assess the role of CRF in mediating sleep is also exemplified in work in humans. Four intravenous injections of human CRF at hourly intervals increased cortisol and reduced both REM and NREM in young healthy male volunteers (Holsboer et al., 1988). By comparison, hourly intravenous administration of cortisol decreased REM and increased NREM and plasma cortisol concentrations (Born et al., 1989; Friess et al., 1994). Together, these studies suggest that the decrease in REM after systemic CRF is mediated by cortisol, whereas the decrease in NREM may be a direct effect of CRF. CRF also appears to contribute to disinhibition of REM sleep and impaired NREM in depression, which is thought to be a stress related disorder. For example, administration of a CRF antagonist to patients with depression decreased REM density and awakenings and increased NREM (Held et al., 2004). Thus, the effects of CRF also may vary with route of administration, potentially with species, and across situations with altered neural functioning.

### CRF and stress-induced hyperthermia

Stress-induced increases in core body temperature can begin within 10 s of the onset of stress induction (Clement et al., 1989; Krarup et al., 1999) and can be as much as 2°C in rats and mice for a variety of stressors including handling stress, exposure to novel environments, and restraint (Briese and De Quijada, 1970; Singer et al., 1986; Clement et al., 1989; Zethof et al., 1994). Anxiolytic drugs can decrease SIH (Vinckers et al., 2009). By comparison, some anxiogenic drugs have minimal effects on SIH possibly due to a maximum limit to the amount temperature may increase (Vinckers et al., 2009) whereas others may reduce or prevent SIH responses (Houtepen et al., 2011).

All of the stress conditions that we examined produced increases in average body temperature relative to time matched Base though there were differences in the time course of the response. Notably, the HC condition, putatively a milder stressor, returned to Base levels more rapidly whereas the conditions involving foot shock (both SES and SIS) had more persisting increases, regardless of whether the mice had received SAL, AST, or CRF.

As we reported before for training with ES alone (Yang et al., 2011b), AST did not produce a significant attenuation of SIH for either SES or SIS trained mice. However, other studies have reported that CRF antagonists attenuate SIH. The CRF receptor 1 antagonists antalarmin and SSR125543A [4-(2-chloro-4-methoxy-5-methylphenyl]-*N*-[(1*S*)-2-cyclopropyl-1-(3-fluoro-4-methylphenyl)ethyl]5-methyl-*N*-(2-propynyl)-1,3-thiazol-2-aminehydrochloride) administered orally or intraperitoneally reduce SIH in rats subjected to isolation stress (Griebel et al., 2002). ICV administration of the broad CRF antagonist αHelCRF in rats also reduced body temperature after exposure to a cage change stressor (Morimoto et al., 1993; Nakamori et al., 1993). Interestingly, central administration of CRF in rats produces an increase in body temperature (Heinrichs et al., 2001; Figueiredo et al., 2010) that is attenuated by AST and antalarmin (Figueiredo et al., 2010).

The reason for the differences compared to our results is not clear, but both isolation and cage change likely are less intense stressors than the footshock paradigm used in our studies. They also likely involve only psychological stress whereas footshock involves both psychological and strong physiological stress which would activate additional neural pathways. Furthermore, though *in vitro* assays indicate that AST is more potent for both CRF1 and CRF2 receptors than is αHelCRF (Hauger et al., 2006), *in vivo* studies in rats suggest that AST may be less potent in preventing some CRF- and stress-induced and anxiety-related behaviors including CRF-induced locomotor activity (Spina et al., 2000). It is also possible that a higher dosage of AST than we used (1.0 μg) may be effective in reducing SIH. For example, a 5.0 μg, but not a 1.0 μg, dosage of AST blocked the reduction in the number of entries into the open arms of an elevated plus maze produced by central administration of 0.5 μg CRF (Spina et al., 2000). Strain or species differences in the central CRF system could also be involved. BALB/cJ mice, the strain used in this study, have differences in their CRF system (Blank et al., 2003) and reactivity to CRF and CRF antagonists compared to C57BL/6J mice (Sanford et al., 2008), thereby suggesting differences in the regulation of sleep by CRF.

### CRF and the functional significance of post-stress sleep

SIH responses have a time course that parallels that of hypothalamic-pituitary-adrenal (HPA) axis activation (Veening et al., 2004; van Bogaert et al., 2006) and both SIH and corticosterone (Shors et al., 1989) are similarly enhanced by ES and IS which are followed by distinctly different alterations in sleep. Fear, as indicated by behavioral freezing, also is very similar in response to contextual reminders of SES and SIS (Machida et al., 2013) or ES and IS alone (Yang et al., 2011a). This suggests that the mere presence of a peripheral stress response and fearful behavior do not dictate, or even predict, the types of alterations in sleep that occur after acute stress. Instead, post-stress sleep appears to be a function of the context in which the stressor was encountered and the emotional and learning processes that were engaged as the animal evaluated and reacted to the situation. In line with the effects of waking experiences on sleep, various lines of evidence suggest that, in turn, sleep plays a significant role in adaptive responding to stress. For instance, sleep disturbances both before (Bryant et al., 2010) and after (Lavie, 2001) significant or traumatic events have been linked to the development of stress-related pathology.

The increases in REM that occur following controllable stress (Sanford et al., 2010) and following fear extinction (Wellman et al., 2008), and the decreases in REM that can occur without recovery following uncontrollable stress (Liu et al., 2003; Sanford et al., 2003c) and associated with learned helplessness (Adrien et al., 1991) suggest that post-stress REM may play a role in adaptive responses to stress. This is also consistent with findings in posttraumatic stress disorder (PTSD) patients that found, in sleep recordings soon after trauma, a more fragmented pattern of REM characterized by shorter average duration REM episodes before shifting stage or awakening in PTSD patients compared to patients without PTSD and a non-traumatized comparison group (Mellman et al., 2002, 2007). There were also a greater number of REM episodes in the PTSD patients than in patients that experienced trauma without developing PTSD. These differences led to the suggestion that intact REM aids in the processing of memory for trauma (Mellman et al., 2002, 2007). Similar hypotheses emphasizing a positive role for REM in mediating the effects of stress have been put forth including suggestions that REM plays an important role in consolidating memories for aversive events and in “decoupling” those memories from their emotional charge (Nishida et al., 2009; Walker, 2009) and that it serves to weaken unwanted memory traces in the cortex (Crick and Mitchison, 1983). Experimental paradigms employing controllable and uncontrollable stressors that produce clear distinctions in post-stress sleep should be useful for determining the role that sleep may have in orchestrating neurobiological changes that promote adaptive behaviors and the role that impaired sleep may play in stress-induced pathologies.

The current results suggest that centrally acting CRF is a significant regulator of post-stress sleep. It also has been linked to stress-related psychopathology. Elevated levels of CRF have been found in the cerebrospinal fluid (Bremner et al., 1997; Baker et al., 1999; Sautter et al., 2003) and plasma (de Kloet et al., 2007) of PTSD patients and elevated CRF coupled with either enhanced negative feedback or downregulated CRF receptors has been hypothesized to play a role in the reduced delta sleep found in patients with PTSD (Neylan et al., 2006). Stress also can be a significant factor in insomnia (Healey et al., 1981; Basta et al., 2007) which is associated with higher activity in the HPA axis and considered to be a disorder of hyperarousal in the central nervous system (Bonnet and Arand, 1997; Vgontzas et al., 2001; Basta et al., 2007). Increased CRF activity figures prominently in hypotheses regarding the pathogenesis of primary insomnia (Richardson and Roth, 2001). Thus, CRF may be a significant mediator of the stress and arousal/sleep systems and their interactions in regulating the outcomes of stressful experiences. This is also suggested by our recent findings that antagonizing CRF in the basolateral nucleus of the amygdala (BLA) prior to training with inescapable shock blocked the reductions in REM that normally occur (Wellman et al., 2013). It also blocked subsequent conditioned reductions in REM without reducing freezing, suggesting that fear memory had been altered, but not blocked. By comparison, inactivation of BLA with the GABAergic agonist, muscimol, blocked the reduction in REM, and reduced freezing (Wellman et al., 2014), suggesting that CRF was working in a region that mediates the memory linkage between fearful behaviors and sleep.

### A note on experimental design

We chose to complete the microinjections of CRF before we conducted studies with AST. This was based on the concern that antagonizing CRF early in the series might alter subsequent stress responses, or the impact of stress on sleep. For that reason, we conducted SAL controls for both CRF and AST to be able to determine whether there were potential carryover effects across the experiment. Based on these controls, the effects of SES and SIS were consistent across treatment days, with the exception of the relative effects of CRF and AST on stress-induced alterations in sleep.

### Conclusion

Post-stress sleep appears to be determined by the types of information available in the stressful context, the animal's processing of that information, and the emotional responses that are induced. Post-stress sleep, in particular REM, also appears to play a role in mediating the positive or negative outcomes of stress, and behavioral experiences during wakefulness likely set in motion the underlying neurobiological processes that guide and enable sleep to mediate the effects of stress. The current results demonstrate that centrally acting CRF is a major regulator of post-stress alterations in sleep that may occur independently of the induction of the peripheral stress response. Work focused on understanding the role that central CRF has in regulating interactions between the stress and arousal/sleep systems should provide insight into the neural processes that are associated with adaptive and non-adaptive responding to stress.

### Conflict of interest statement

The authors declare that the research was conducted in the absence of any commercial or financial relationships that could be construed as a potential conflict of interest.
